# The Role of Adaptive Goal Processes in Mental Wellbeing in Chronic Pain

**DOI:** 10.3390/ijerph20021278

**Published:** 2023-01-10

**Authors:** Tara Swindells, Joanne Iddon, Joanne M. Dickson

**Affiliations:** 1 Psychology Division, School of Arts & Humanities, Edith Cowan University, Joondalup 6027, Australia; 2 Department of Clinical Health Psychology, Mersey Care NHS Foundation Trust, Southport L34 1PJ, UK; 3 Exercise Medicine Research Institute, Edith Cowan University, Joondalup 6027, Australia

**Keywords:** chronic pain, mental wellbeing, goal flexibility, goal tenacity

## Abstract

Chronic pain, experienced as pain persisting for three months or longer, is associated with risk of poor mental health and disability. Research has implicated adaptive goal processes as important to mental wellbeing in a range of populations. However, research has rarely assessed these mechanisms in relation to pain characteristics and mental wellbeing in chronic pain populations. This study aimed to examine the potentially mediating roles of goal flexibility and goal tenacity in the relationships between pain intensity and pain interference and mental wellbeing among individuals with chronic pain. Community members who self-identified as experiencing non-cancer chronic pain (*N* = 315) completed an online self-report survey on goal tenacity, goal flexibility, mental wellbeing, pain intensity, and pain interference. Unexpectedly, pain intensity was not significantly related to mental wellbeing, when controlling for pain interference. However, pain interference was directly and significantly associated with mental wellbeing. Both goal flexibility and goal tenacity mediated the relationship between pain interference and mental wellbeing, whilst controlling for pain intensity. The results provide support for the protective role of adaptive goal processes in mental wellbeing in those with chronic pain and highlight the importance of pain interference in relation to mental wellbeing.

## 1. Introduction

Chronic pain is defined as pain that persists beyond the expected healing time, typically for three months or longer [1]. It is a relatively common condition, impacting approximately 20% of the population in Australia and internationally [2,3]. In addition to medical and physical implications, chronic pain can have far-reaching impacts on an individual’s employment, lifestyle, and mental health [4,5]. It is well documented that chronic pain is associated with elevated risk of psychological distress [6]. Both greater pain intensity (i.e., how much pain is experienced) and pain interference (i.e., the impact of pain on daily functioning) have been independently associated with elevated depressive symptoms and lower mental wellbeing in pain populations [7,8]. Notably, emerging research suggests that goal motivational processes can be protective against pain and mental ill-health and may in fact promote mental wellbeing [9,10]. Given the prevalence of chronic pain in the population, and that it is typically considered a life-long condition, it is important that research continue to investigate potentially adaptive goal processes that may promote mental wellbeing in the context of pain [11]. 

Goals are cognitive representations of desired future states or outcomes that people strive toward [12]. Goals are fundamental to the human experience and psychological wellbeing because they drive individual activity and give meaning to people’s lives [12]. Chronic pain is considered a significant, threatening, and aversive life stressor that interferes with individual functioning and, consequently, valued activities and personal goals [13]. As a result of pain, individuals may not have the psychological and/or physical capacity to engage in activities and behaviours that are consistent with attaining their personal goals. The way an individual with chronic pain reconciles their threatened valued goals can have significant implications on their experience of pain and their mental wellbeing [10,14]. 

The Dual Process Model of coping presents two ways that individuals can cope when valued goals have been threatened or blocked [15]. The model posits that goal tenacity, a form of assimilative coping, refers to persistently striving to achieve a desired goal under difficult circumstances such as pain [15]. This style of coping involves efforts by the individual to alter their situation so that they can attain their goals. For example, individuals might seek out medical intervention such as surgery to relieve their pain or utilise medication to help them cope with their pain. Comparatively, goal flexibility, an accommodative style of coping, refers to adjusting goal pursuits in response to changing circumstances and setbacks [16]. An accommodative style of coping is characterised by reappraisal and involves reorienting from previous, now unattainable goals to more achievable ones. For example, an individual may come to accept that the total eradication of their pain will not be possible, or they may refocus onto other valued areas of their life that are less impacted by pain to meet their valued needs. Although goal tenacity and goal flexibility represent two distinct ways of coping, they are not mutually exclusive and can coexist. However, the Dual Process Model suggests that while a goal is still achievable, assimilative coping is optimal. When a goal becomes unattainable, moving from primarily assimilative coping to accommodative coping is considered most adaptive [16]. 

Past research has demonstrated how these goal processes are related to mental health in the context of chronic pain [8,14]. However, limited research has investigated goal flexibility and goal tenacity in relation to both psychological distress and characteristics of pain (e.g., the intensity of pain and pain-related disability). One study by Schmitz et al. found that goal flexibility moderated the relationships between pain intensity and pain-related disability and depression in individuals with chronic pain, therefore providing a buffer against psychological distress [17]. Further, goal flexibility was found to predict less pain intensity and perceived disability [17]. Conversely, goal tenacity did not moderate the relationship between pain intensity and pain-related disability and depression and did not predict better pain outcomes [17]. However, greater goal tenacity and greater goal flexibility were both related to less psychological distress [17].

Although assimilative and accommodative coping are theoretically complementary, there are mixed findings related to the usefulness of tenacious goal pursuit in response to chronic health conditions [18]. Pursuing important life goals is associated with greater mental wellbeing, psychological adjustment, and purpose in life in those with chronic pain [14,19,20]. Further, disengagement from valued personal goals has been associated with greater rates of negative rumination [14]. However, other research suggests that failure to disengage from unattainable goals can negatively impact on an individual’s psychological adjustment [21]. Theoretically, unyielding tenacious goal pursuit may result in a reliance on potentially unhelpful pain control behaviours that are counterproductive and exacerbate distress [8,22].

Regardless of the ‘goal pursuit dilemma’ present in the literature, both higher goal tenacity and goal flexibility have been associated with increased mental wellbeing in various populations who have experienced significant changes in functioning, including those with neurological disability, multiple sclerosis, and older adults [23,24,25]. Recent research has also highlighted that positive goal engagement is associated with increased mental wellbeing in individuals with chronic pain [9]. Positive goal engagement refers to the ability to identify and orient oneself towards personally meaningful goals by drawing upon one’s own inherent resources and strengths. This research implicated the protective role of positive goal engagement in chronic pain, with positive goal engagement partially mediating the relationships between pain characteristics and improved mental wellbeing [9].

Despite relevant theory, the potential mediating roles of goal flexibility and goal tenacity in the relationships between pain characteristics and mental wellbeing in chronic pain remain unexplored. It is arguable that variations in goal tenacity and goal flexibility may help to explain why some individuals with chronic pain experience greater mental wellbeing than others. As such, this study aims to investigate the potential mediating roles of goal flexibility and goal tenacity in the relationships between pain characteristics (i.e., pain intensity and pain interference) and mental wellbeing among individuals with chronic pain. In this study, pain intensity refers to the sensory dimension of pain severity, while pain interference refers to the impact of pain on daily functioning [26].

**Figure 1:**
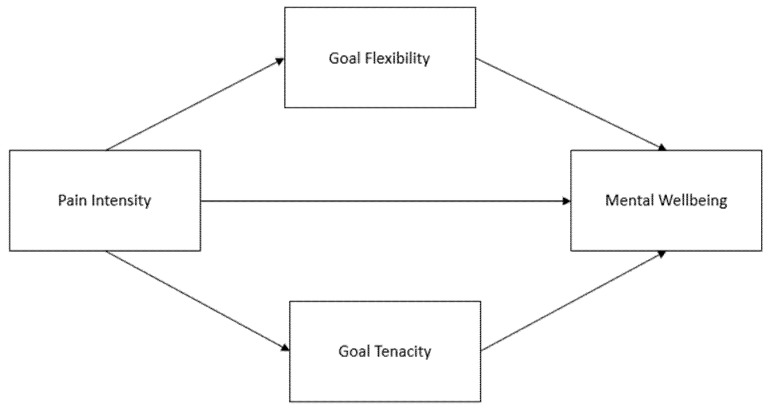
Hypothesised mediation model examining the relationship between pain intensity and mental wellbeing, mediated via goal flexibility and goal tenacity.

**Figure 2:**
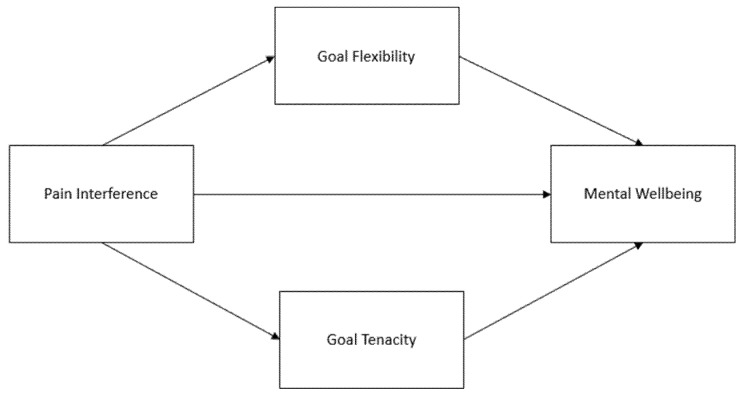
Hypothesised mediation model examining the relationship between pain interference and mental wellbeing, mediated via goal flexibility and goal tenacity.

Specifically, it is hypothesised that in Model One, there will be: (1) a significant direct negative relationship between pain intensity and mental wellbeing, (2) a significant direct positive relationship between goal flexibility and mental wellbeing, and (3) a significant direct positive relationship between goal tenacity and mental wellbeing. Further, as illustrated in Figure 1, it is hypothesised that: (4) goal flexibility and goal tenacity will each indirectly mediate the relationship between pain intensity and improved mental wellbeing. It is hypothesised that in Model Two there will be: (5) a significant direct negative relationship between pain interference and mental wellbeing and, as illustrated in Figure 2, that (6) goal flexibility and goal tenacity will each indirectly mediate the relationship between pain interference and improved mental wellbeing. 

## 2. Materials and Methods

### 2.1. Research Design

This study used a correlational, cross-sectional design to explore the relationships between pain intensity and pain interference (predictor variables), goal flexibility and goal tenacity (mediator variables), and mental wellbeing (outcome variable).

### 2.2. Participants

Participants were adults living in the community who reported experiencing non-cancer physical pain for three consecutive months or longer, in line with contemporary definitions of chronic pain [27]. Consistent with existing literature, chronic pain was differentiated from cancer pain due to the differing prognoses and treatments available, and the unique psychosocial factors associated with cancer pain (e.g., concern for mortality) [28]. Other inclusion criteria included living in Australia and being fluent in English.

To detect a small–medium effect size between the direct and indirect pathways, as informed by previous research [14], using a bias-corrected bootstrap analysis, Fritz and Mackinnon recommend a sample of at least 148 participants [29]. The study adequately met a priori power calculations, with a final sample of 315 participants (*M age* = 40.63, *SD* = 12.31), of which 88.9% identified as a woman, 7.9% identified as a man, and 3.1% identified as non-binary or in a different way. The majority of participants (89.8%) reported that they heard about the survey through social media. Participants were also recruited from pain management service providers such as physiotherapy clinics and psychology clinics (3.2%), Chronic Pain Australia newsletters and website (2.2%), and word of mouth (4.8%).

To meet inclusion criteria, participants needed to be 18 years old or over, live in Australia, and self-identify as experiencing non-cancer physical pain for 3 months or longer. Of the total sample, 93% reported that they had a specific pain condition, and 90.2% reported that they had received a formal diagnosis for their pain. The most commonly reported pain conditions were fibromyalgia and varying forms of arthritis. A list of the pain conditions reported by participants and the prevalence of these conditions are presented in Table 1. The mean length of time participants reported experiencing their pain was 12.61 years (*SD* = 10.81). Of the participants, 92.7% indicated that they currently took medication to relieve their pain.

### 2.3. Measures

#### 2.3.1. Demographic Information

Demographic items were used to capture information on age, gender, and where the individual heard about the survey. Participants were also asked about the nature of their pain, including if they had a specific pain condition, if they had received a formal diagnosis, duration since pain onset, and if they used analgesic medication.

#### 2.3.2. Pain Characteristics

The Brief Pain Inventory Short Form (BPI-SF) was used to measure the intensity of participants’ pain and the impact of their pain on their daily functioning [30]. Used extensively in clinical, non-clinical, and research settings, the self-report measure is composed of 11 0-to-10 numeric rating scales. The pain intensity subscale comprises 4 items asking participants to rate their pain from 0 (no pain) to 10 (pain as bad as you can imagine) at its present, least, most, and average in the past 24 h. The pain interference subscale contains 7 items related to areas of functioning, with participants asked to rate their pain from 0 (does not interfere) to 10 (completely interferes). Items relevant to each subscale were calculated to create a mean score, with higher scores indicating greater pain intensity or pain interference, respectively. 

Past research has shown that the BPI-SF has demonstrated excellent internal consistency across the intensity and interference subscales in individuals with non-cancer chronic pain, with α = 0.84 and α = 0.91, respectively [9]. In the present study, internal consistency was strong, with α = 0.86 for intensity and α = 0.88 for interference. Support for the two-factor structure among individuals with non-cancer pain indicates the measure’s strong construct validity [31]. 

#### 2.3.3. Goal Tenacity and Goal Flexibility

The Tenacious Goal Pursuit and Flexible Goal Adjustment Scale (TEN/FLEX) was used to measure two types of adaptive goal processes; tenacious goal pursuit (TGP; e.g., “When faced with obstacles, I usually double my efforts”) and flexible goal adjustment (FGA; e.g., “In general, I am not upset very long about a missed opportunity”) [15]. Each subscale comprises 15 items. Participants rate statements from 1 (strongly disagree) to 5 (strongly agree). Scores for each subscale were summed, with higher scores indicating greater goal tenacity and goal flexibility. Past research has shown that the TGP and FGA subscales report good internal consistency in populations with chronic pain, with α = 0.80 and α = 0.81, respectively [14]. In the current sample, each subscale demonstrated similarly strong internal consistency, with α = 0.86 for the TGP and α = 0.86 for the FGA. 

#### 2.3.4. Mental Wellbeing

The Warwick–Edinburgh Mental Well-Being Scale (WEMWBS) was used to measure mental wellbeing [32]. The WEMWBS contains 14 positively phrased item-statements to assess positive aspects of psychological functioning (e.g., I have been feeling relaxed). Participants rate the item statements from 1 (none of the time) to 5 (all of the time). Items were summed together, with higher scores reflecting a higher level of mental wellbeing.

The WEMWBS has strong internal consistency in populations with chronic non-cancer pain (α = 0.93) [9]. Good criterion validity has been illustrated in a sample of the general adult population, with lower scores on the General Health Questionnaire which screens for general mental health disorders associated with higher scores on the WEMWBS (*r* = −0.62, *p* <.001) [33]. Internal consistency was excellent in this study’s sample (α = 0.91).

### 2.4. Procedure

Ethical approval was obtained from the Edith Cowan University’s Human Research Ethics Committee (HREC: 2022–03174-Swindells). Participants were recruited by responding to study advertisements displayed at participating pain management services (e.g., physiotherapy clinics, psychology clinics) and online advertisements promoted through Australian chronic pain advocacy and charity groups and on social media. A link to the online survey, hosted by Qualtrics, directed interested participants to the study information letter that outlined the study aims, participation requirements, voluntary participation, right to withdraw, risks, benefits, and data storage arrangements. Participants provided informed consent before proceeding to the survey by indicating that they understood what participation involved. Following the demographic items, participants were then presented with the WEMWBS, TENFLEX, and BPI-SF. The BPI-SF was presented last to negate any potential negative priming effects through completing items related to pain symptoms and impairments [34]. The survey took approximately 15 min. Upon completing the survey, participants were thanked and presented with debriefing information, which included local support service numbers specific to chronic pain (e.g., Chronic Pain Australia) and general counselling services (e.g., Lifeline). Participants who wanted to enter the prize drawing to win one of two $50 Coles/Myer vouchers, and/or be provided with a summary of study results, were directed to a separate webpage where they could provide their contact details.

### 2.5. Data Analysis

Data analysis was conducted using SPSS v28. Preliminary analyses assessed descriptive statistics and bivariate correlations to examine associations between study variables and to identify any potential confounding variables. Two hypothesised mediation models were then tested using the PROCESS macro (model 4) [35]. The first mediation model assessed the predicted indirect effects of pain intensity on mental wellbeing via goal flexibility and goal tenacity, while controlling for pain interference and age. The second mediation model assessed the predicted indirect effects of pain interference on mental wellbeing via goal flexibility and goal tenacity, while controlling for pain intensity and age. The corresponding predictor variable was controlled for in each model to ensure that any variance explained by the independent variable and mediators could not also be accounted for by the control variable. Indirect effects and 95% bias corrected confidence intervals were estimated using 10,000 resampling draws [35].

## 3. Results

We first report the preliminary analyses including data screening, descriptive statistics and correlational analyses, followed by the mediational analyses to test the main hypotheses. 

### 3.1. Preliminary Analysis

A missing value analysis indicated minimal missing data (<1%) at random. Subsequently, expectation maximisation was used to impute missing data [36]. All parametric assumptions were met, indicating that mediational analyses using PROCESS v4.1 could be meaningfully interpreted [35]. The data were absent from any extreme outliers [36]. Inspection of Mahalanobis distances identified three cases that exceeded the critical χ^2^ for df = 4 (at α = 0.001) of 18.47. However, given that Cook’s distance was less than 1 for these multivariate outliers, they were not deemed a concern [36]. 

Descriptive statistics and the correlations between the main study variables are reported in Table 2. As expected, both pain intensity and pain interference were significantly negatively correlated with mental wellbeing, such that greater pain intensity and greater pain interference were associated with lower mental wellbeing. Pain intensity and pain interference were significantly positively correlated. Of the adaptive goal processes, only goal flexibility was significantly correlated with pain intensity, such that greater goal flexibility was associated with lower pain intensity. Both goal flexibility and goal tenacity were significantly negatively correlated with pain interference, such that greater goal flexibility and greater goal tenacity were associated with lower pain interference. As expected, greater goal tenacity and goal flexibility were significantly positively correlated with greater mental wellbeing. Age was significantly positively correlated with mental wellbeing and goal flexibility, with older participants more likely to report greater mental wellbeing and greater goal flexibility. Hence, age was included as a covariate in the mediation analyses. Independent samples *t* tests indicated no statistically significant differences between genders in scores on any of the main study variables (all *p*’s > 0.05). Analgesic medication use was not significantly correlated with any of the main study variables (all *p*’s > 0.05).

### 3.2. Model Testing

#### 3.2.1. Model One: Pain Intensity, Goal Flexibility and Goal Tenacity, and Mental Wellbeing

**Figure 3:**
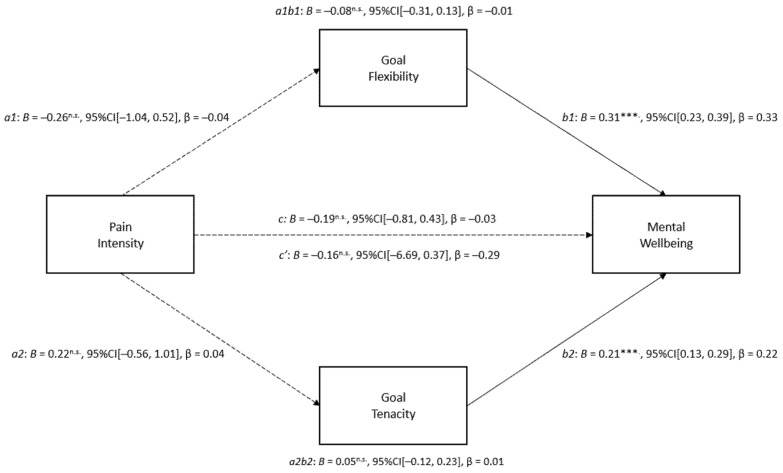
Results of Model One mediation analysis. Note: The solid lines signify significant pathways, and the broken lines signify non-significant pathways. *** *p* < 0.001. ** *p* < 0.01, * *p* < 0.05. n.s. = non-significant.

Model One tested the direct and indirect effects of pain intensity on mental wellbeing via goal tenacity and goal flexibility, whilst controlling for pain interference and age. In combination, pain intensity, goal tenacity, goal flexibility, pain interference, and age accounted for a statistically significant 54% of variance in mental wellbeing, R^2^ = 0.55, F (5, 309) = 75.15, *p* < 0.001. According to Cohen’s conventions, this is a large effect (f^2^ = 1.22). Unstandardised (B) and standardised regression coefficients (β) and 95% confidence intervals for Model One are presented below in Figure 3. Counter to prediction, there was no direct relationship between pain intensity and mental wellbeing (c’ = −0.16, *p* = 0.56). There was also no direct relationship between pain intensity and goal flexibility (B = −0.26, *p* = 0.52) or pain intensity and goal tenacity (B = 0.22, *p* = 0.58). As predicted, both goal flexibility (B = 0.31, *p* < 0.001) and goal tenacity (B = 0.21, *p* < 0.001) were uniquely positively related to mental wellbeing. Counter to prediction, there was no indirect effect of pain intensity on mental wellbeing via goal flexibility (ab = −0.08, LLCI/ULCI = 0) or goal tenacity (ab = 0.05, LLCI/ULCI = 0). Indirect pathways accounted for 16.88% of the total effect of pain intensity on mental wellbeing; however, it is important to note that the total effect of the model was not significant (c = −0.19, *p* = 0.55). In summary, neither goal flexibility nor goal tenacity significantly explained the relationship between pain intensity and mental wellbeing.

#### 3.2.2. Model Two: Pain Interference, Goal Flexibility and Goal Tenacity, and Mental Wellbeing

**Figure 4:**
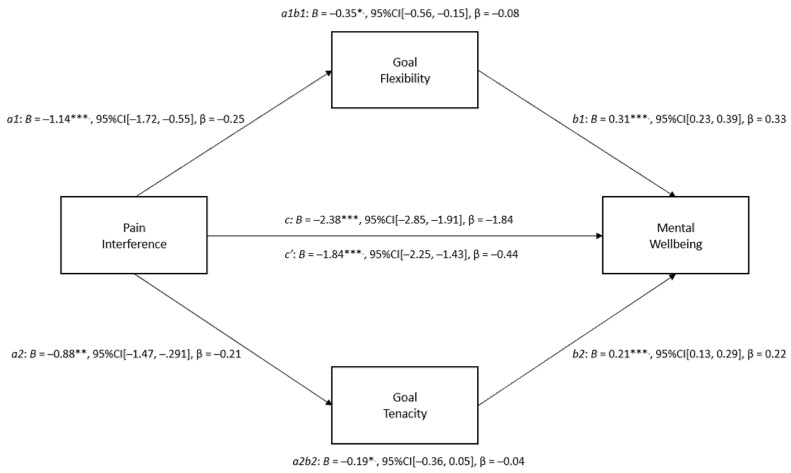
Results of Model Two mediation analysis. Note: The solid lines signify significant pathways, and the broken lines signify non-significant pathways. *** *p* < 0.001. ** *p* < 0.01, * *p* < 0.05.

Model Two tested the direct and indirect effects of pain interference on mental wellbeing via goal tenacity and goal flexibility, whilst controlling for pain intensity and age. Unstandardised (B) and standardised regression coefficients (β) and 95% confidence intervals for Model Two are presented below in Figure 4. As predicted, there was a significant direct negative relationship between pain interference and mental wellbeing (c’ = −1.84, *p* < 0.001). Both goal flexibility (B = −1.14, *p* < 0.001) and goal tenacity (B = −0.88, *p* < 0.01) were also uniquely negatively related to pain interference. As anticipated, an indirect effect of pain interference via goal flexibility (ab = −0.35, LLCI/ULCI ≠ 0) and goal tenacity (ab = −0.19, LLCI/ULCI ≠ 0) predicted a significant proportion of unique variance in mental wellbeing. The total effect of the model was significant (c = −2.38, *p* < 0.001), with the indirect pathways accounting for 22.60% of the total effect of pain interference on mental wellbeing. In summary, both flexibility and tenacity (while controlling for each other in the one model) were each shown to uniquely and significantly explain the relationship between pain interference and mental wellbeing.

## 4. Discussion

In recent times, understanding and treatment of chronic pain has transitioned from a predominantly biomedical model to a biopsychosocial approach that acknowledges the role of psychological processes in the pain experience [37]. As such, research examining psychological processes that may help to manage pain and promote mental wellbeing in those with chronic pain is useful, particularly given the condition’s enduring nature and association with psychological distress [6]. This study aimed to investigate the potentially mediating roles of the adaptive goal processes of goal flexibility and goal tenacity in the relationships between pain intensity and pain interference and mental wellbeing among individuals with chronic pain. Unexpectedly, pain intensity was not directly or indirectly associated with mental wellbeing, and neither goal flexibility nor goal tenacity mediated this relationship. However, as expected, pain interference and mental wellbeing were directly and indirectly associated, with both goal flexibility and goal tenacity indirectly mediating the relationship. The results of this study offer support for the protective role of adaptive goal processes in maintaining mental wellbeing in those with chronic pain. The results also highlight the importance of pain interference in the pain experience and its potential impact on mental wellbeing. Further, findings provide additional evidence for the relevance of utilising asset-based constructs and measures to explore adaptive psychological processes in the context of chronic illness [9].

As expected, and consistent with past research, there was a significant negative bivariate correlation between pain intensity and mental wellbeing in this study [8,14]. However, once the covariance of pain interference was accounted for, pain intensity and mental wellbeing were no longer related. Similar to findings by Iddon et al., these results suggest that it may be the pain interference on daily life, rather than the intensity of the pain, that drives the relationship between chronic pain and lower mental wellbeing [9]. It is possible that individuals can find ways to maintain their mental wellbeing when their pain intensity is high, so long as it does not interfere with important aspects of their daily life such as mobility, work, and relationships with others. However, when pain starts to interfere with an individual’s engagement in meaningful daily activities, individuals may start to experience distress and reduction in subjective mental wellbeing [13]. This premise is consistent with personal goal theory, which posits that distress increases when valued activities and future outcomes are blocked [38]. 

There are several reasons why the results of this study relating to pain intensity and mental wellbeing may counter those of previous studies [8,17]. Firstly, the literature on chronic pain tends to be limited in its methodology to assessing pain intensity and pain interference and their relationships with psychological constructs independently, paying less attention to their co-occurrence. Accounting for both pain intensity and pain interference together recognises the multidimensional experience of pain. In addition to findings by Iddon et al., similar results were observed in a study that investigated the relationships between depression, pain intensity, and pain interference in acute spinal injury when both pain characteristics were accounted for [9,39]. Cuff et al. found that pain interference had a greater bearing on symptoms of depression than pain intensity alone [39]. 

The results of this study provide additional support for the protective role of goal motivational processes in maintaining mental wellbeing in those with chronic pain [9,14,17]. Specifically, results from Model Two support the idea that goal flexibility and goal tenacity may buffer the negative emotional impacts of pain interference on mental wellbeing. The findings suggest that when pain interference is high and goal flexibility and goal tenacity are low, individuals are more likely to report lower mental wellbeing. However, when goal flexibility and goal tenacity are high, individuals are more likely to report higher mental wellbeing, despite the functional impact of their pain. By engaging in self-regulatory activities to help individuals continue to strive toward their goals (e.g., taking pain medication, engaging in physiotherapy) and adjusting their goals to their new circumstances, it is plausible that individuals may find ways to offset the functional impact of their pain. These findings are useful when considering the pragmatics of chronic pain treatment. Often the functional impact of pain (e.g., impact on walking, work, and mobility) may not be changeable; however, the way in which people find ways to cope with their pain and its impacts could be. 

Notably, in this study, both goal tenacity and goal flexibility appeared to buffer the impact of pain interference on mental wellbeing, when simultaneously controlling for each goal mechanism within one model, conflicting with some previous findings [17,19]. However, similar to findings by Ramírez-Maestre et al., goal flexibility appeared to be the most effective in protecting individuals’ wellbeing against goal losses and constraints associated with pain interference [14]. This was indicated by the fact that the indirect effect of pain interference on mental wellbeing via goal flexibility was almost double that of goal tenacity. These findings are consistent with Brandtstädter’s Dual Process Model in that, although both goal processes may be helpful to wellbeing, reappraising one’s goals when they no longer match with personal capabilities and circumstances is most adaptive [15]. Given that goal flexibility involves adapting and adjusting to difficulties as they arise, it is possible that this goal process may tap into the broader higher-order ability of psychological flexibility, whereby individuals tend to notice and respond to situations in functional, open, and effective ways in pursuit of long-term goals [40]. Comparatively, psychological inflexibility has been associated with behavioural avoidance in situations that might induce pain, which might perpetuate and maintain distress [40]. In addition to adaptive persistence, goal tenacity might elicit rigidity in some individuals, which may, in part, account for the different effect sizes.

### 4.1. Implications

The results suggest that pursuing goals and re-appraising them in response to setbacks may be beneficial to the mental wellbeing of those in the broader community who are experiencing pain. As study participants were primarily recruited through social media and websites, it would be valuable to summarise these results utilising a psychoeducational approach and web-accessible format to promote the potential benefits of personal adaptive goal pursuit. The findings have implications for informing public health policy developments and public health campaigns focused on, for example, positive self-care and messaging related to pain management, as well as informing ongoing effective developments in clinical practice.

In addition to providing support for Brandtstädter’s Dual Process Model, this study also highlights the relevance of psychological processes in the management and treatment of chronic pain, consistent with current biopsychosocial approaches [15]. Specifically, the findings support the potential usefulness of psychological interventions that utilise goal setting and goal-directed action to promote and sustain mental wellbeing in chronic pain [41,42]. For instance, an intervention that has indicated promising results in chronic pain populations is Solution Focused Brief Therapy (SFBT) [41]. SFBT for chronic pain invites clients to imagine and describe their personally meaningful hopes for the future, identify their own internal strengths and resources, and recognise small signs of success happening in an effort to improve self-efficacy, wellbeing, hope, and coping [42]. Identifying an individual’s tendency to engage in different ways of coping represented by goal flexibility and goal tenacity could be helpful clinically for all health professionals within the multidisciplinary team when facilitating collaborative conversations with clients and utilising interventions based on these findings within the chronic pain context. 

### 4.2. Limitations and Future Research

Given the cross-sectional nature of this study, the results cannot establish causal inferences or the direction of effects between pain interference, goal flexibility and goal tenacity, and mental wellbeing. For example, it is plausible that greater mental wellbeing may predict greater engagement in goal tenacity and goal flexibility. Longitudinal research is warranted to examine engagement of goal processes over time and how they may impact mental wellbeing in chronic pain populations. Future research would also benefit from the inclusion of other measures of emotional functioning and wellbeing, as well as examining how types of goals (e.g., health and recovery goals compared to goals from other spheres of life) may relate to individuals’ pain and mental wellbeing. Like much of the existing literature, this study relied on self-report data on goal processes, which can be subjective and represent preferences of coping rather than actuality. Ecological momentary assessment of goal-directed behaviour could provide greater insight into the interrelationships of adaptive goal processes, wellbeing, and pain experiences. More objective measures of pain interference, such as clinician-rate measures of disability, would also be useful. Lastly, the findings of this study highlight the importance of considering pain intensity and pain interference together in future research, to account for the multidimensional nature of chronic pain.

## 5. Conclusions

Overall, this study highlights the important role of the goal processes of goal flexibility and goal tenacity in the relationship between pain interference and mental wellbeing in those with chronic pain. Although there was no apparent relationship between pain intensity and mental wellbeing in this study, goal flexibility and goal tenacity mediated the effects of pain interference on mental wellbeing, appearing to provide a buffering effect. Additionally, the level of pain interference appeared to have greater implications for the mental wellbeing of individuals with chronic pain than did pain severity intensity itself, an important fact for clinicians and researchers to consider. The results of this study are promising and suggest goal processes may be an effective way to help individuals sustain mental wellbeing when dealing with chronic pain.

## Figures and Tables

**Table 1:** Frequency of pain conditions reported by participants.

Diagnosis	n	%
Ankylosing Spondylitis	12	2.5
Arthritis	86	17.6
Arthritis type unspecified	24	4.9
Osteoarthritis	37	7.6
Psoriatic Arthritis	10	2.0
Rheumatoid Arthritis	15	3.1
Back Pain	44	9.0
Chronic Regional Pain Syndrome	11	2.2
Complex Regional Pain Syndrome	14	2.9
Ehlers–Danlos Syndrome	21	4.3
Endometriosis	24	4.9
Fibromyalgia	142	29.0
Headaches	26	5.3
Neuropathic Pain	14	2.9
Pelvic Pain	12	2.5
Scoliosis	10	2.0
Other	73	14.9

Participants could indicate more than one condition.

**Table 2:** Descriptive statistics and correlations between model variables.

Variable	Descriptive Statistics	1	2	3	4	5	6	7	8
	*M (SD)*								
1. Formal diagnosis ^a b^	-	-	0.01	−0.03	−0.02	−0.05	0.85	0.01	0.02
2. Age	40.63 (12.31)		-	0.35 **	0.02	0.02	0.25 **	−0.04	0.15 **
3. Time experienced pain (years)	12.61 (10.18)			-	0.10	0.05	0.12 **	0.06	0.10
4. Pain Intensity	5.31(1.51)					0.61 **	−0.20 **	−0.09	−0.38 **
5. Pain Interference	6.57 (2.01)					-	−0.27 **	−0.18 **	−0.59 **
6. Goal Flexibility	44.96 (9.06)						-	0.32 **	0.56 **
7. Goal Tenacity	43.75 (8.58)							-	0.40 **
8. Mental Wellbeing	38.50 (8.41)								-

^a^ 1 = Yes, 2 = No. ^b^ Correlations between dichotomous and continuous variables are point bi-serial correlations. * *p* < 0.05. ** *p* < 0.01.

## Data Availability

Anonymous data were collected and saved in an SPSS datafile. This SPSS dataset will be deposited at Edith Cowan University’s data repository, and accession numbers will be made available upon request.
